# Notices

**Published:** 1868-04

**Authors:** 


### NOTICES.

MERCHANTS' AND BANKERS' ALMANAC for 1868, published at the office of the Bankers' Magazine and Statistical Register 46 Pine st., New York, sold for $2, and sent post paid at same price. Every intelligent Merchant Banker and business man will find in this beautifully bound 8vo of about 250 pp. statistics of Monthly Prices for Forty Years, of Cotton, Wool, Pork, Flour, Wheat, Corn, Oats, Hops, Iron, Copper, Coal, Coffee, Molasses, Sugar, &c. Annual Product of Wheat, Corn, Rye, Potatoes, in each State, 1866. List of New Publications on Banking, Finance, &c. List of Banks, Bankers, Savings Banks, in Canada. Daily Price of Gold, 1863–1867. Price of Consols Annually, 1750–1866. Fluctuations in 150 Stocks, Bonds, &c., 1867. Progress of Railroads, 1834–1866. Clearing-House Statistics of New York and London. Prices of 80 Staple Articles, Monthly, 1867. List of 2,000 Cashiers. Prices Monthly of American Securities, London, 1867. Engravings of New Bank Buildings, with a List of 1,650 National Banks: 270 State Banks. List of 1,400 Bankers in the United States. List of 1,200 Brokers and Bankers in New York City. List of 320 Savings Banks in New England and New York. List of Bankers in Great Britain, Europe, Asia, Australia, South America, &c. List of 600 Insurance Companies in the United States. It will be a valuable book of reference for years to come.

THE YOUNG MAN SETTING OUT IN LIFE: By William Guest, F.G.S., published by the American Tract Society, 150 Nassau street, New York, 151 p.p., 16 mo., sent post-paid for forty cents. This is a small book, a London reprint, and we advise every parent who reads this, who has a son about setting out in life, to send for a copy and in consequence of its intrinsic value give its notice more space than we could otherwise do. The merchant prince, christian gentleman and philanthropist, George H. Stuart of Philadelphia, rejoices in its republication and says of it, the lectures are short, pointed, truthful, eloquent and comprehensive. Wm. E. Dodge, President of the New York Young Men's Christian Association says, "It is able, practical, sensible and direct. It was brought to this country by my brother Rev. D. Stuart Dodge, who has now returned from Syria. He felt it met the wants of young men peculiarly and was anxious for its publication here." The Tract Society adds, " The price of this book is thirty cents in cloth, and 20 cts. in paper ; but the committee of the Society, wishing to aid young men's christian associations in reaching the youth, with valuable truth, will make a grant equal to their purchase of this and other small books in paper, as well as of tracts"—address S. W. Stebbins, Depository 150 Nassau St. New York, or 13 Cornhill, Boston, Mass. Among the subjects are, How will you use life? Power of character; Grandeur of Destiny ; How you may reach it.

LIVING LONG. To live long and healthfully in civilized life, good food properly cooked is of first importance, or as the Talmudic maxim has it ; " In the pot in which you cook, you will yourself be cooked." Prof. Blot, pronounced Blow, has published through the Appletons a Hand Book of practical cookery, for ladies and professional cooks, containing the whole science and art of preparing human food, with the motto " If ye be willing and obedient, ye shall eat the good of the land," 478pp. 12mo $2. and sent by mail for same price. As the courteous Professor is a friend of ours and his book ought to be in the household of all our readers, we give a notice at length of its contents &c., being persuaded that good cooking is an element of health and long life ; ali of us eat too much and too fast, and it will take a thousand years to educate, even the intelligent, to a wise action in these regards, it will perhaps be a more direct way of antagonizing these errors to lead to a better manner of cooking, because if food is well cooked, the effects of the two vices above named will be modified, to a considerable extent. The Professor in his preface says, " The man who does not use his brain to select and prepare his food, is not above the brutes which take it in its raw state. It is to the body, what education is to the mind. Good and well prepared food beautifies the physique as a good education beautifies the mind. A cook book cannot be used to advantage if theory is not blended with practice. Five items of advice are given to cooks. 1st, Make use of everything good. 2d, Waste nothing, however little. 3d, Have no prejudices. 4th, Be careful, clean, punctual. 5th, Remember you have agreed to faithfully perform your daily duties for a certain consideration. Among the list of contents are directions for soups, sauces, farces and garnitures, fish, beef, mutton, veal, pork, poultry, game, vegetables, eggs, maccaroni, rice, sweet dishes, pastry, bills of fare, &c. The science and art of cooking may be divided into ten parts viz. Baking, Boiling, Broiling, Frying, Mixing, Roasting, Sautéing, Seasoning, Simmering and Stewing. Boiling is the most abused branch in cooking. Some things are boiled for economy, which ought to be prepared in another way. Boil slow ; fast boiling makes steam faster, but does not cook faster, because water cannot be hotter than boiling hot, while by the fast boiling the flavor is evaporated and cannot be brought back. Sautéing is akin to frying, the latter requires fat enough to immerse the article cooked, but to sauté it, use only fat enough to keep it from being scorched ; vegetables, omelets &c., should be sautéd, not fried, and require a brisk fire, the quicker cooked thus, the better.

TASTING is the most difficult and delicate part of seasoning ; in this only two senses are used, smell, and taste, and without these no one can cook properly."

Prof. Blot has not lived a score of years almost in New York for nothing. He knows somewhat of the individuals New Yorkers employ at fifteen dollars a month to dig their graves ; and he has become a philosopher. Two directions to cooks suggest volumes.

HAVE NO PREJUDICES. Season to the taste of your employer, and not your own ; but to that end, the first thing the employer is to do in taking a new cook is to instruct how to season to suit themselves, and require the cook to keep to that point. O you tempest tossed and tormented New York housekeepers ! hav'nt you found that it is as much trouble to create a cook as to induce one to abandon her prejudices and to do things your way and not her own. If you show them to-day ; they will reply Yes'm ; and to-morrow, without the slightest possible compunction they will prepare it in the same way they have always done, as if they had never heard of you. Now my Dear Lady Reader let me tell you something worth knowing. When you employ help about your house, give them distinctly to understand, that you will try to keep them and show them and do what you can to make their place easy, but if they require to be showed or told the same thing the third time, you will dismiss them, however valuable they may be, and then do it. Patience and forbearance beyond that point is no virtue ; because you pay them to do what you want them to do and not to please themselves. But this last paragraph is our own, not the Professor's, and we will only add, there is an almost universal misapprehension, and at the same time a contemptuous turning up of the nose at the mention of "French cookery," arising however from a misunderstanding of what it really is ; there ought to be no other in the wide world, for its essential features are,

First, To make inferior food palatable and healthful.

Second, To utilize every scrap and waste not a morsel.

Third, Variety of preparation, so that if from poverty or otherwise the same kind of food mainly has to be eaten, it may not be grown tired of.

True French cookery is to use very little grease, very little butter and no spices. For example, instead of using butter for everything in cooking, as most of our wasteful and extravagant cooks do, the " drippings " as they are called are carefully preserved and used in such a way as to answer better than butter. Now surely no rational person can object to these principles of cookery. Nine cooks out of ten in New York who advertise for places as superior cooks seem to labor under the impression that the more butter, the more brandy. the more sugar and the more aqua-fortis are put in any given dish, the better it is. The fact is, four-fifths of the miserable ignorant, stupid, dirty, outlandish persons whom we are obliged to employ to cook for us and to kill us, are not fit to cook for a cow ; they are stubborn, wilful and have but one principle of conduct, to get the utmost farthing of the wages promised for the least possible work for the maintenance of their places ; and it is very certain, that many of them waste and break and give away of their employer's property, more than their wages would amount to. A pretty effectual method of abolishing these latter practices is to have a fair understanding in the beginning that one half the value of the breakage is to be deducted from their wages ; and that nothing must be given away by them at the door but money ; a different practice encourages recklessness in washing dishes, which becomes a habit, not easily broken, when they begin to keep house themselves. To allow them to sell the "Soap fat" is giving them a premium to take your butter for which you are paying sixty-five cents a pound for this February 1868 and sell it at three cents a pound ; while to give away your food at the door to their countrymen is to encourage beggary, idleness, thriftlessness and theft. The truth is a very large share of the worthlessness and incompetence of our domestics is owing to the previous unfitness of persons to be mistresses. When will we get to the point of educating our daughters to the sphere for which they are destined, the presidency of their own households and the economizing of what husbands toil and strive for, family provisions and comforts. Talk about women's rights, and women's voting! and then in the next breath twatter about their being overworked ; to remedy that it is proposed to give them more to do ; for to vote intelligently, the state of the country must be studied and kept before the mind ; legislative debates must be read ; governors' messages and reports of heads of departments must be understood and analyzed, and many other things looked into. Woman's throne is at the family fireside, at the head of the table ; in the social circle ; when she leaves these, she makes the first step towards her degradation and instead of being the angel of the household, she becomes the common property of the mob and "Ichabod" will be written of her the moment she mounts the platform or presents herself at the poles ; and when the glory is departed ; when woman ceases to be the Queen of our hearths and hearts—then we'll step out, will go to the country for a few days and "imbibe" for the remainder of the journey, for there would be no sort of use in living any longer.

HEALTHFUL HEAT.—To have no coal dirt or coal gas in our parlors ; to have a cheerful blazing fire on a level with the floor, from a wide fireplace, sending out a heat to the middle of the room, using but two scuttlefulls a day in the very coldest weather, equal to sixty pounds nett or less than a ton a month, being twenty-five cents a day when coal is seven dollars a ton ; but the average cost from Nov. 1st to May 1st would be about fifteen cents a day ; the first scuttlefull of thirty pounds will last until One p. m., and if another is then thrown on it will last until late bed time, without having to use the poker once, during the whole day, hence there is no dust or ashes floating about the room and no ashes to be taken up in the morning, and to know how such desirable things are done, using either wood, peat, hard or soft coal, let any reader call at any hour of the day at our house number Two West Forty-third street, New York, one door west of Fifth Avenue, and he will be welcomed to see the gratifying sight ; it certainly is the most delightful and the most healthful artificial heat in the world ; and of the great number of persons who have been induced to purchase the Low Down Grate of Dixon & Sons, Philadelphia, we have not known a single one who at the end of five, six and eight years' use does not continue to be delighted with it.

POTATOE BAKER, patented by A. Reid, Buffalo, N. Y. It is arranged to occupy the place of a boiler on the top of a stove, requiring but little fuel and the work is quickly done.

*Reasons why "Reid's Patent Baker" is spoken so highly of by those who have used it.* "It will bake potatoes over a small fire in summer. It will bake them on any kind of a stove with a griddle hole. It will bake them in the morning as quickly as you can boil a kettle of water. It will bake them in the evening when the fire is quite low. It does away with the heating of the oven specially to bake potatoes. It has a shelf which can be raised or lowered according to the heat of the fire. It saves fuel all the year round. It saves heat in summer. It saves time. It enables you to enjoy the best esculent ever placed upon the table just when you please. It saves health. How frequently do we hear it said, 'I'd like baked potatoes for breakfast, if I could only get them in time.' No excuse when you have a Patent Potato Baker in the house. Never need to wait."

"THE GALAXY" is published monthly at 39 Park Row, New York at $3.50 a year. March is a continuation of "Stephen Lawrence, Yeoman, by Mrs. Edwards; John Bright at Home; Worthless Laurels; Some celebrated Shrews; A Deserted Plantation; The secret history of a subsidized organ; Words and their uses; Elder Knapp the revivalist; How Samirande was caught; Southern Troubles and their remedy; Manners of the day," &c. THE GALAXY is well conducted; its pages are filled with useful, instructive, entertaining and practical matter and deserves to be classed with the first monthlies in the land. The article on "Shrews," to wit. Mrs. Job, Mrs. Socrates, the Immortal Xantippee, Mrs. Sir Thomas More, Mrs. Parquier, Countess of Shrewsbury, Mrs. Albert Durer, Mrs. Berghen, Mrs. William Shakespeare, the Wife of "Coke upon Littleton," of Dr. Andrew Bell, of Ainsworth's Latin Dictionary, of George Whitfield, good old John Wesley, the wife of the great Duke of Marlboro, and a great many others in the course of nine pages; by Frank M. Ballard; single numbers will be sent post-paid for thirty cents. If all these ladies were actual scolds, no doubt they had satisfactory reasons for the same, and Mr. Ballard ought to have had gallantry enough to have ferreted out their reasons in all the cases, whereas he has only done it in one instance, that of the celebrated Mrs. Xantippee, who has quite as wide a reputation as her husband Socrates, and we will leave it to any lady reader if Mrs. X. does not merit general sympathy, for Socrates said he married her, not for love or money, but as a means of self discipline; he spent his time in talking on the sidewalk to any one who would listen to him, and such he called his scholars; he did nothing towards the support of his family, for his wife had to support him; besides, he had an ugly face, goggle eyes, flat nose, thick lips, bald head, squat figure, clumsy walk, his clothes were ragged, and he delighted in walking about town, barefoot. Scaliger said his wife was a hectic fever, which death only could cure. When good old John Wesley's at long last left him after twenty years of domesticities, he simply wrote "I

did'nt send her away, and I won't go to bring her back." Now this plainly showed that the old fellow loved "circuit riding" more than he did his wife,

"And sic a wife as Wesley had!
"He did na care a button for her,"

and she didn't care a button for him and so they agreed to disagree. We don't know who Mr. Frank Ballard is, but no doubt he is a dried up, crusty old bach, and because no body of any account would take him, he takes the grim pleasure of revenge in spending many weeks and months in trying to make out that it is not best for a man to marry, giving out the intimation that the reason of it is, women are cross and fault-finding and revengeful; in all cases giving a one sided version; get out Frank Ballard you never had a wife or daughter or sister or mother, you just sprouted up in some of the Jersey flats or the Dismal Swamp and will doubtless dry up and wither away into your original nothingness. We advise every man who thinks he has a scolding wife to send for the March number of THE GALAXY, but as a large edition will be wanted, we advise the Messrs. Church to borrow the *Tribune* presses for a month or two; otherwise they will fall short of the demand, so perverse are men, and so incapable of justly appreciating woman's worth.

INGROWING TOE NAIL.—Make a bridge of muslin from the big toe to the next toe but one, and allow this middle toe to rest on the muslin bridge; this effectually removes the pressure against the big toe, and the parts eventually get well. See also Health Tract 64.

DEBT.—Many a generous, manly-hearted fellow has been discouraged into suicide; others have been plunged into drunkenness; others still have abandoned themselves into hopeless helplessness from the crushing incubus of debts incurred which they cannot liquidate. Let the reader be warned by the experience of others, and heed the advice of Horace Greeley:

"Hunger, cold, rags, hard work, contempt, suspicion, unjust reproach, are disagreeable, but debt is infinitely worse than them all. And if it had pleased God to spare either or all of my sons to be the support and solace of my declining years the lesson which I should have more earnestly sought to impress upon them is—"Never run into debt! Avoid pecuniary obligation as you would pestilence or famine. If you have but fifty cents, and can get no more for a week, buy a peck of corn, parch it and live on it, rather than owe any man a dollar."

THE DESPOTISM OF DRUNKENNESS is strikingly exemplified in the Senate:

"One gentleman who was an exemplary member of the Congressional Temperance Society a year ago, astonished and appalled the boarders at Willard's, one morning last week, by entering the breakfast-room in his night-shirt at about 10 A. M.; another was taken home in a handcart, not many Sundays since; while another, over whose conversation all the ladies were, a year ago, saturating their pocket-handkerchiefs, (need I say he is a distinguished Senator from the West?) has been, for a week or more, confined to his room and a diet of pickled cabbage, to help him over a prolonged carouse, terminating in the deliriums."

If Members of Congress, with a nation's eyes upon them, cannot have the moral courage to break off from the habit of drinking, how is it possible for the masses to do so? But are not excessive tea-drinking and coffee-drinking, and gluttony, in the same line? All are the slaves of low animal appetites.

MADNESS.—"In this very place, when full of mirth and joy she was called by her mother (oh, so fondly!) Lolotte—by her father, Lotchen. Then she, too, grown fair and saintly, was called the Princess Charlotte. Afterwards, when a blooming bride, she became the Archduchess Charlotte. Last of all, the Empress of Mexico! And now she is nothing more than the poor maniac—the helpless, hopeless lunatic."

Reader! art thou sane? Are you sure of it? Then "walk softly before God," and be thankful always, lest thou fall into the same condemnation; and as a physical means of avoiding insanity, be busy, moderate your ambition, live temperately, and get abundant, regular and natural sleep.

MARRIAGE AND CROPS.—It is a statistical fact, that in the twelve years ending with 1865, in England and Wales, the number of marriages was in direct proportion to the price of wheat; the cheaper the wheat, the greater the number of marriages. It may be thus expressed in figures: out of 1,000 marriageable couples, 808 were married when the price of wheat was 64s.; 830 when wheat brought 52s., and 854 when wheat was 42s. This would seem to show that social enjoyment and virtue and true prosperity attend low prices, and that high prices beget extravagance, high living, debauchery and every evil work.

WORRIED to DEATH.—This is a very common expression with a metaphorical meaning; but many a time, alas! it is literally true, especially so with the over-sensitive—the too high-strung. But it is often an unnecessary result, arising from idleness giving time to brood over trifles, or from the wicked and weak-minded habit of getting into a worry about trifling things. I once knew a lady to cry because it rained before she could have some work finished around her splendid city mansion. All of us should accustom ourselves to take things by their smooth handle, remembering that it has been wisely said, the chief secret of comfort lies in not suffering trifles to vex one, and in prudently cultivating an overgrowth of small pleasures, since very great ones are let on long leases.

ENGAGING MANNERS.—It is the duty of all, young and old, rich and poor, to dress as tidily and as becomingly as their tastes and circumstances will permit, and in every situation, and in all companies, to exhibit the deportment characterized by kindness, courtesy and consideration. A nameless one has written:

"There are a thousand pretty, engaging little ways, which every person may put on without running the risk of being deemed affected or foppish: The sweet smile, the cordial bow, the earnest movement in addressing a friend, the inquiring glance, the graceful attention which is so captivating when united with self-possession—these will insure us the good regards of even a churl. Above all, there is a certain softness of manner which should be cultivated, and which, in either man or woman, adds a charm that almost entirely compensates for lack of beauty, and inestimably enhances the latter, if it does exist.

IDLENESS.—No man was made to be a loafer; it is a crime against oneself, a crime against society, a crime against the Merciful One who has enacted the universal law, "In the sweat of thy face thou shalt eat bread," with the added injunction, "Be diligent in business." In this connection Mrs. Dall has truthfully and forcibly written:

"There is no greater enemy to body and soul than idleness. unless it is that public sentiment which compels to idleness. Thousands, and tens of thousands, have fallen victims to it. The woman who will not labor, rich or honored though she be, bends her head to the inevitable curse of heaven. This curse works in failing health, fading beauty, broken temper, and weary days. Let her never fancy that, being neither wife nor mother, she is exempt from the law. She cannot balance that decree of God by the foolish customs of society, or the weak objections of kindred. Disease, depression, moral idiotcy or inertia, follow an idle life. He who never rests has made woman in his image; and health, beauty, force and influence, follow in the steps of labor alone."

SMOKING.—Mr. Parton, who devotes his life to literary labor, and who was himself a smoker once, says:

"The vast majority of smokers—seven out of every ten—can, without the least danger or inconvenience, cease smoking totally and for ever. I was myself a smoker for thirty-years, but I am now free; I can work better and longer than before; I have less headache; I have a better opinion of myself; I enjoy exercise more, and step out much more vigorously; my room is cleaner; I think I am better tempered, as well as more cheerful and satisfied; it did not pay to smoke, but most decidedly it pays to stop smoking."

There is a better and more manly way than this: omit the liquor, have the moral courage to plant your foot down and say "I"ll never smoke again," and let that be that be end of it; he who leans upon brandy for any thing leans upon a spear which, in the breaking, may pierce him to the heart. Only babies and weak-minded fools want help to do right. When old Dame Dobson was advised to try the water cure for lhe "rumatiz," she said, "why, that's no new thing; it's as old as the deluge, and even then it killed more'n it cured. Brandy cure is ditto.

CLERICAL LONGEVITY.—Of fifty-two clergymen whose ages are given at death, one-half were over seventy years; of these two were over ninety, showing, as we have often, that studiousness does not necessarily abridge human life.

